# Evaluating motivational interview quality using large language models and hidden Markov models

**DOI:** 10.1186/s12888-025-07391-1

**Published:** 2025-10-01

**Authors:** Kyungho Lim, Young-Chul Jung, Byung-Hoon Kim

**Affiliations:** 1 Department of Psychiatry, Yonsei University College of Medicine, 50-1 Yonsei-ro, Seodaemun-gu, Seoul, 03722 Republic of Korea; 2 Institute of Behavioral Sciences in Medicine, Yonsei University College of Medicine, Seoul, Republic of Korea; 3 Department of Biomedical Systems Informatics, Yonsei University College of Medicine, Seoul, Republic of Korea; 4 Institute for Innovation in Digital Healthcare, Yonsei University, Seoul, Republic of Korea

**Keywords:** Motivational interview, Large language model, Hidden markov models, Interview analysis, Interview quality assessment

## Abstract

**Background:**

Motivational Interviewing (MI) is a counseling approach that promotes behavior change by eliciting “change talk” and minimizing “sustain talk.” Traditional methods for assessing MI quality, such as manual coding, are labor-intensive, subjective, and difficult to scale. This study introduces an automated framework integrating large language models (LLMs) and Hidden Markov Models (HMMs) for evaluation of MI session quality.

**Aims:**

This study evaluates the effectiveness of an LLM-HMM framework in predicting MI session quality and examines motivational state transitions in high- and low-quality sessions.

**Method:**

A dataset of 40 MI sessions was analyzed. Client utterances were classified and numerically scored by an LLM based on their intention toward or away from change. With HMMs, we used these scores to examine the motivational state transitions across each session. Differences between high- and low-quality sessions were quantified by comparing transition matrices using Frobenius norms. Statistical significance was assessed via a permutation test. Predictive performance was evaluated using logistic regression with leave-one-out cross-validation (LOOCV), where transition matrix elements served as independent variables and interview quality as the dependent variable.

**Results:**

High-quality MI sessions exhibited fluid transitions between motivational states, whereas low-quality sessions showed persistence in resistance-oriented states. A statistically significant difference in transition matrices was observed between session groups (*p* < 0.001). The framework achieved a mean LOOCV accuracy of 0.80, demonstrating strong predictive performance in identifying MI session quality.

**Conclusions:**

This study presents a scalable, objective alternative to manual MI evaluation. Future applications may include real-time therapist support, training, and prognosis prediction, pending further validation on field-collected data.

**Supplementary Information:**

The online version contains supplementary material available at 10.1186/s12888-025-07391-1.

## Background

MI is a client-centered counseling approach that elicits and strengthens motivation for change [1–5]. A key mechanism of MI lies in its ability to evoke “change talk” while addressing and reducing “sustain talk.” Change talk refers to client statements that indicate a desire, ability, reason, or commitment to change, while sustain talk reflects ambivalence or resistance, focusing on maintaining the status quo [2, 6]. The interplay between these types of talk has profound implications for behavioral outcomes, as research consistently links the frequency and quality of change talk to successful behavior change [7]. Conversely, unchecked sustain talk may hinder progress and signal ineffective engagement during MI sessions.

Evaluating change talk and sustain talk is essential in MI because these client utterances reflect underlying motivational states and guide the counselor’s strategy [1, 6]. Change talk often signals readiness for action, providing the counselor with opportunities to reinforce momentum toward positive behavioral changes [2, 6]. On the other hand, sustain talk highlights ambivalence or barriers to change, prompting the counselor to explore these obstacles empathetically while steering the conversation back toward change [2, 6]. Failure to adequately identify and respond to these cues may result in missed opportunities to facilitate progress, underscoring the importance of accurate and objective evaluation methods [6].

The effectiveness of MI is highly dependent on interview quality, which is traditionally assessed through manual coding schemes like the Motivational Interviewing Treatment Integrity (MITI), or surveys such as Clinical Evaluation of MI and Client Evaluation of MI (CEMI) [8, 9]. While reliable, these manual evaluations are resource-intensive, subjective, and prone to variability [8]. Coders must identify, categorize, and score client utterances, which demands substantial training and time investment. Additionally, human evaluators may introduce bias or inconsistencies, particularly when assessing ambiguous utterances or balancing complex dynamics between change and sustain talk.

Recent advances in LLMs have shown promising results in understanding nuanced conversations and detecting subtle cues indicating changes in mental health [10–14]. These advancements have facilitated the development of virtual counselors equipped with various clinical interviewing skills, including the ability to conduct MI [9]. However, the evaluation of these systems has so far relied on manual methods that are labor-intensive and have a risk of bias or inconsistencies. Therefore, the demand for automating the assessment of MI quality to overcome the limitations of manual evaluations and provide a more consistent and scalable solution has never been greater.

Generative pre-trained Transformer (GPT) is an architecture of LLM that has demonstrated remarkable capabilities in natural language understanding and generation [15]. Their ability to classify complex utterances into predefined categories makes them well-suited for analyzing counseling sessions [16]. This proposed LLM’s ability to analyze counseling sessions suggests the potential not only for accurately evaluating clients’ talk types in MI but also for assessing the actual impact of MI on clients’ readiness for change. To effectively model these client attitude shifts within a counseling session, computational tools such as HMMs are particularly relevant. HMMs, which are probabilistic models widely used for time series analysis involving unobservable hidden states, have demonstrated their effectiveness in psychological studies and counseling session analysis [17–19]. In these applications, HMMs have been used to identify latent states underlying observable behaviors, enabling a deeper understanding of complex interactions and their implications. For example, studies have shown that modeling transitions between client states can predict treatment outcomes and inform therapeutic adjustments [20].

HMMs also allow for the quantification of hidden motivational states and their transitions, capturing the temporal dynamics of change talk and sustain talk over the course of an MI session [21]. This pairing enables a nuanced analysis that extends beyond traditional methods, revealing patterns and insights that might otherwise remain obscured. For instance, HMMs can model how a client transitions between states of ambivalence, commitment, or resistance, offering counselors actionable insights into the effectiveness of their strategies.

Building on this foundation, we propose a proof-of-concept framework that integrates LLMs and HMMs for the objective and quantitative evaluation of MI. This framework leverages the ability of LLMs not only to classify client utterances but also to score the inclination of each utterance on a numerical scale. These scores, which reflect movement toward or away from change, serve as critical inputs for HMMs to model motivational state transitions. By analyzing these transitions alongside the numerical strength of client inclinations, we aim to provide a comprehensive assessment of interview quality and identify distinguishing features of high- and low-quality MI sessions. The primary goal of this study is to examine the feasibility and potential utility of this framework for identifying meaningful motivational dynamics and differentiating between high- and low-quality sessions.

In doing so, this study addresses several critical gaps in the current literature. First, it advances the understanding of motivational dynamics in MI by providing a quantitative framework to analyze the interplay between hidden motivational states that are not explicitly observable during the interview or within the transcript. Second, it introduces a scalable and automated alternative to manual coding, evaluation, and feedback traditionally performed by human professionals, thereby reducing the resource burden associated with conventional methods. Finally, it contributes to the broader field of behavioral and psychological research by demonstrating the utility of LLMs and HMMs in capturing and interpreting complex human interactions.

## Methods

**Fig. 1:**
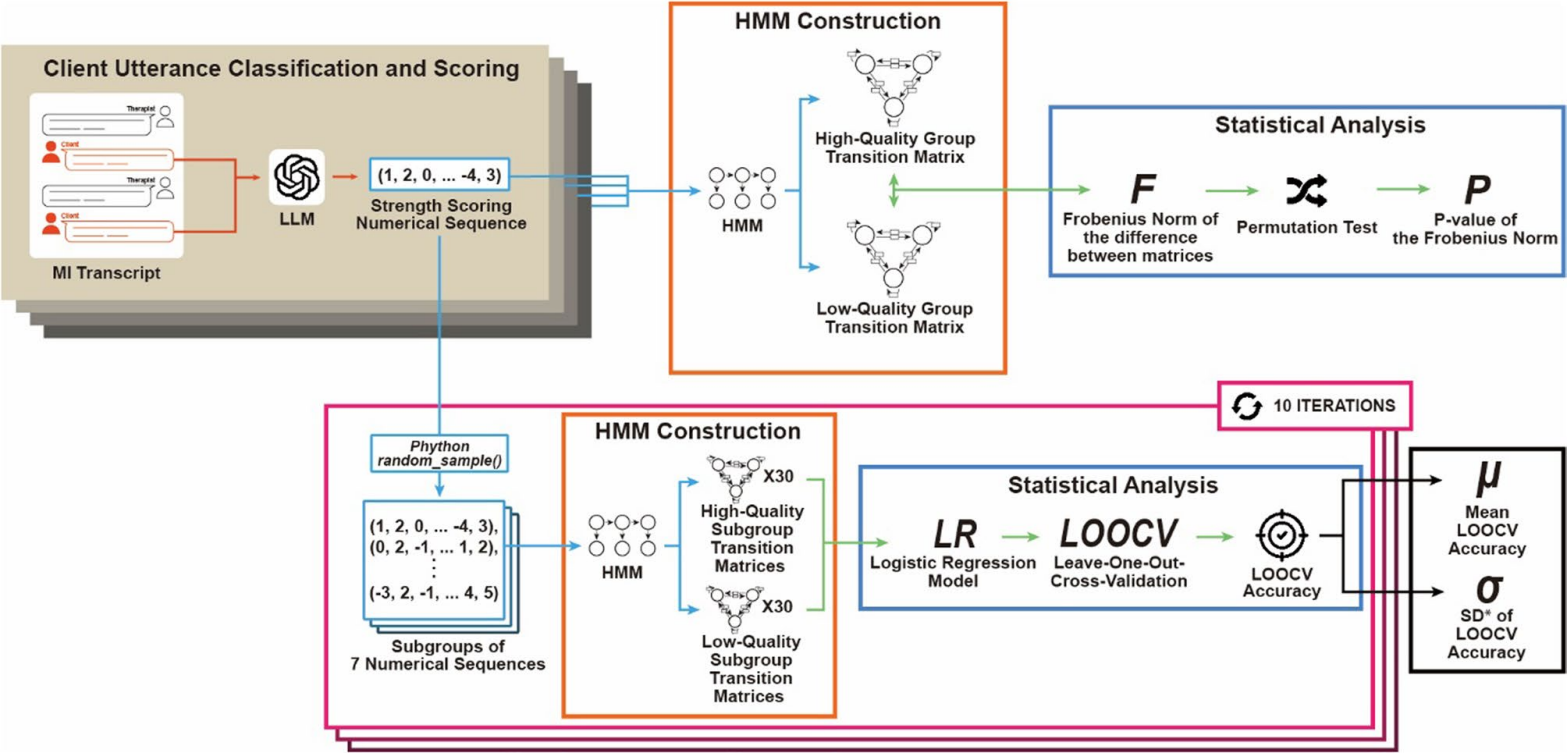
Schematic representation of the study workflow. The figure outlines the sequential processes involved in the evaluation of MI interview quality. Utterance classification and scoring using GPT-4o, *SD: Standard deviation

Figure 1. provides a schematic overview of the workflow, illustrating the processes of data collection, utterance classification and scoring using LLMs, and motivational state transition analysis via HMMs. Each component is described in detail in the following subsections.

### Data collection

For MI transcripts for evaluation of this study, we used “Anno-MI”, a freely accessible public dataset of professionally transcribed and expert-annotated therapy dialogues [22]. Each session was labeled as either high-quality or low-quality based on the titles, descriptions, or commentary provided by the original video creators (e.g., “MI-Good Example,” “How NOT to do Motivational Interviewing”) [22]. The utterance-level annotations were produced by ten expert annotators affiliated with the Motivational Interviewing Network of Trainers (MINT) [22]. Each transcript was annotated by a single expert therapist, and these labels were used directly as ground-truth inputs for model training. To assess inter-annotator agreement, a shared subset of seven transcripts was independently labeled by all ten annotators. No adjudication or consensus process was applied to these overlapping annotations. Fleiss’ kappa scores on this subset were 0.74 for therapist behavior and 0.47 for client talk type, indicating substantial and moderate agreement, respectively [22]. The dataset consisted of 113 sessions. After excluding sessions which had noises that obscured the meaning of the entire interview, we got 30 high-quality sessions and 10 low-quality sessions, ensuring a representative sample for analysis. Noisy data, such as meaningless repetitions of certain characters (e.g., ‘n/an/an/an/an/a’) or duplicate lines missing client talk type annotations that rendered them unsuitable for modeling, were excluded on rule-based regular expression criteria. Specifically, we filtered out the samples that contained repetitive “n/a” patterns or duplicate lines in the original dataset. All preprocessed transcripts were subsequently reviewed by a board-certified psychiatrist to ensure the accuracy and completeness of the final dataset.

To support transparency and reproducibility, we have released the cleaned and structured version of the AnnoMI dataset used in our study at: https://github.com/kyungholim627/annomi-cleaned-lmmhmm.

### Utterance classification and scoring

The first step in the analysis involved using an LLM to classify client utterances. GPT-4o was selected as the primary LLM for this study and was prompt-instructed to categorize each utterance into one of three motivational categories.

1. Towards Change: statements reflecting a desire, ability, reason, or commitment to change.
2. Neutral: statements that are ambiguous or do not clearly reflect a stance on change.
3. Away from Change: statements reflecting resistance, ambivalence, or a focus on maintaining the status quo.

The LLM performed two key tasks in this step of the process: utterance classification and strength scoring. Via LLM, each client utterance was classified as change talk, sustain talk, or neutral. After that, each utterance was assigned a strength score ranging from − 5 to + 5, reflecting the degree of movement towards or away from change. Positive scores indicated movement towards change, while negative scores indicated resistance. Neutral utterances were scored 0. The output of this classification-scoring process was a numerical sequence of scores, representing the client’s motivational trajectory. These numerical scores served as the input for the Hidden Markov Model (HMM) in the subsequent analysis, allowing motivational trajectories to be inferred based on utterance-level dynamics.

Because the AnnoMI dataset includes only categorical utterance labels (e.g., change talk, sustain talk), and does not contain human-provided numeric strength annotations, the strength scores used in this study were generated solely by the LLM without ground-truth supervision. However, the scoring was guided by prompt instructions aligned with established MI coding principles, and the outputs were reviewed and confirmed by a board-certified psychiatrist to ensure consistency, plausibility, and clinical validity.

To simulate a real-time interview environment, the LLM evaluated each utterance based solely on the information available up to that point in the interview. To enhance the consistency and effectiveness of this process, we implemented the experiments utilizing the LangChain v0.0.313^1^ tools, including `ConversationBufferMemory`, `ChatPromptTemplate`, and message-based inputs like `HumanMessage` and `AIMessage` [23]. `ConversationBufferMemory` was used to maintain context across the session, ensuring that prior exchanges informed the analysis of subsequent utterances. `ChatPromptTemplate` was leveraged to construct prompts that embedded the “spirit” and general principles of MI as system messages, providing the LLM with a clear framework for understanding the motivational dynamics of the session [24]. This structured approach allowed the LLM to analyze data consistently and reliably, converting string data into message-based formats that aligned with the nuances of MI. The complete prompt instructions are provided in the Supplementary material.

### HMM construction

HMMs were used to model the underlying motivational states that generated the observable sequences. The observable sequences consisted of numerical strength scores (ranging from − 5 to + 5) assigned to each client utterance by the LLM in 2.2 *Utterance classification and scoring step*. The model parameters included “Hidden States,” which were three states representing motivational dynamics: “Towards Change,” “Non-Determined,” and “Away From Change.” The likelihood of transitioning from one hidden state to another was obtained as “Transition Probabilities”. Each hidden state was identified and interpreted based on the mean of its emission distribution, which reflected the central tendency of these strength scores (produced by the LLM) associated with that state. These scores informed the classification of states into motivational dynamics, such as ‘Towards Change,’ ‘Non-Determined,’ and ‘Away from Change.’ The initial hidden state of each interview transcript was assigned by the previous prompt-instructed LLM. The Baum-Welch algorithm was used to estimate the model parameters, and the Viterbi algorithm was applied to decode the most likely sequence of hidden states for each session. Transition matrices summarizing the probabilities of moving between states were extracted for further analysis. The HMM and the Viterbi algorithm were implemented using Python based on the `hmmlearn v0.3.3` library. The library provided the foundational framework to parameterize hidden states, transition probabilities, and observation likelihoods. The Viterbi algorithm, integral to decoding the most probable sequence of hidden states, was directly applied using built-in functionalities of `hmmlearn`.

### Statistical analysis

To evaluate the performance of the LLM in categorizing clients’ talk types, we compared the LLM’s classifications with human annotator responses provided in the Anno-MI dataset. We calculated both accuracy and Cohen’s Kappa to assess the model’s performance. To determine whether the transition matrices differed significantly between the high- and low-quality groups, we calculated the Frobenius norm of the element-wise difference to quantify the difference between matrices [25, 26]. This norm captures the overall structural dissimilarity between the matrices by aggregating the squared differences across all corresponding entries. A permutation test was conducted to assess the statistical significance of the observed difference [27]. In this test, the group labels of the sessions were randomly shuffled 1,000 times to generate a null distribution of Frobenius norms under the assumption that the two groups were not different. The *p*-value was calculated as the proportion of permuted differences that were greater than or equal to the observed difference.

### Quality prediction

To explore the predictive potential of the HMM-derived transition matrices in distinguishing interview quality, we employed a logistic regression model combined with leave-one-out cross-validation (LOOCV). Due to the limited length of individual MI sessions, we could not reliably estimate HMM parameters on a per-session basis. Short interviews often failed to provide sufficient data for the HMM to converge or produced sparse and unstable transition matrices. To address this, we created 30 subgroups within each quality category with each subgroup comprising 7 sessions randomly sampled from their respective quality pools. Sessions could appear in multiple subgroups, but each subgroup was constructed as a distinct sample.

For each subgroup, a transition matrix was derived using the HMM, and the flattened elements of the transition matrix were used as independent variables in the logistic regression model. The interview quality (high-quality or low-quality) of the subgroup served as the dependent variable. To prevent an overlap between training and testing data, we performed LOOCV at the subgroup level. In each fold, one subgroup was held out for testing, and the model was trained on the remaining data, with no data from the test subgroup used in training. To comprehensively assess predictive performance, we reported the mean LOOCV accuracy, ROC AUC, and class-wise precision, recall (sensitivity), specificity, and F1 scores for both high-quality and low-quality sessions. ROC curves were also plotted using model-predicted probabilities to visualize threshold-independent model performance, and a confusion matrix was computed to illustrate class-level prediction patterns.

To Further evaluate the robustness of the classification framework, we conducted an additional validation analysis using the same procedure described above, but with a dataset that preserved the original class distribution of the Full sample. Specifically, we sampled 30 subgroups from high-quality sessions and 10 subgroups from low-quality sessions (each comprising 7 sessions), maintaining the original ~ 3:1 ratio of high- to low-quality interviews.

To contextualize the added value of our structured modeling pipeline, we also conducted a comparative baseline experiment using a large language model (GPT-4o) prompted directly to classify the entire session as high- or low-quality. This approach bypasses the utterance-level scoring and HMM modeling, simulating a direct single-shot inference scenario. We provided identical motivational interviewing background information and instructions in the prompt for each session and repeated the procedure across 10 runs to account for variation. We then evaluated the resulting predictions using accuracy, class-wise recall(sensitivity), specificity, F1 score, and a confusion matrix.

### Comparison with other LLM models

To examine the impact of model architecture and scaling on the evaluation performance of our framework, we experimented our framework with the following LLMs as a replacement for the GPT-4o utterance evaluation: GPT-4, Llama-3 70B, and Gemma 7B. These models were chosen to represent variations in parameter sizes and training optimizations, enabling exploration of their ability to assess MI interview quality. Each model was evaluated using the same dataset and framework described earlier.

## Results

**Fig. 2:**
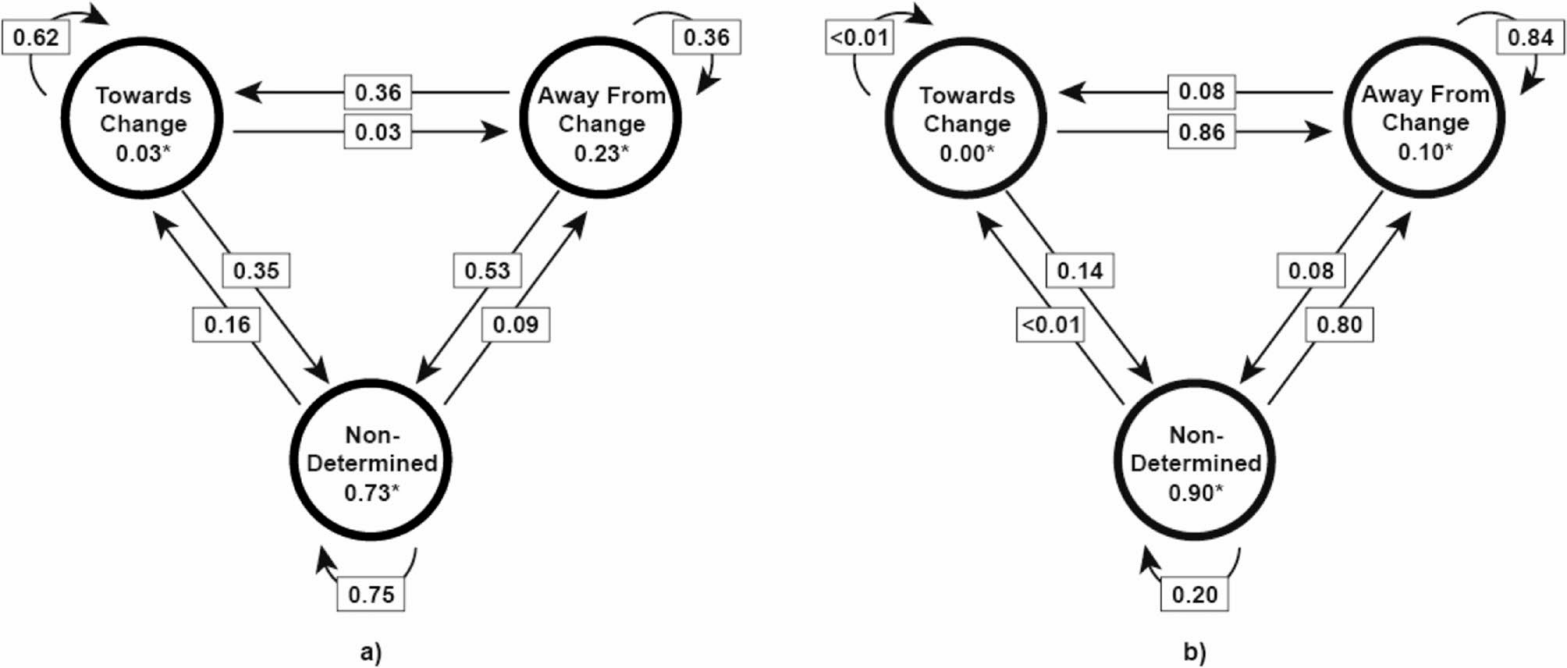
(**a**) GPT-4o produced High-Quality Group HMM and transition matrix: (**b**) GPT-4o produced Low-Quality Group HMM and transition matrix: Asterisked values denote the probability of starting in each state at the beginning of the session. Arrow labels represent the transition probabilities between states during the interview

GPT-4o successfully categorized client utterances, producing sequences that were consistent with expert annotations, showing 0.74 accuracy and Cohen’s Kappa score of 0.53 with expert annotations. Considering the fact that inter-annotator agreement (IAA), using Cohen’s Kappa, reported in the previous studies for tasks involving MI ranged from 0.40 to 0.70, these sequences served as reliable input for the HMM analysis [22, 28–31]. The model design and the transition matrix of the estimated final model for the high-quality and low-quality groups are shown in Fig. 2.

The observed Frobenius norm of the difference between these matrices was 1.54. The *p*-value from the permutation test was < 0.001, indicating a statistically significant difference. High-quality sessions were characterized by balanced transitions among states, reflecting dynamic motivational engagement. In contrast, low-quality sessions showed a dominance of transitions leading to and persisting in the “Away from Change” state, indicating a lack of progress or engagement.

**Fig. 3:**
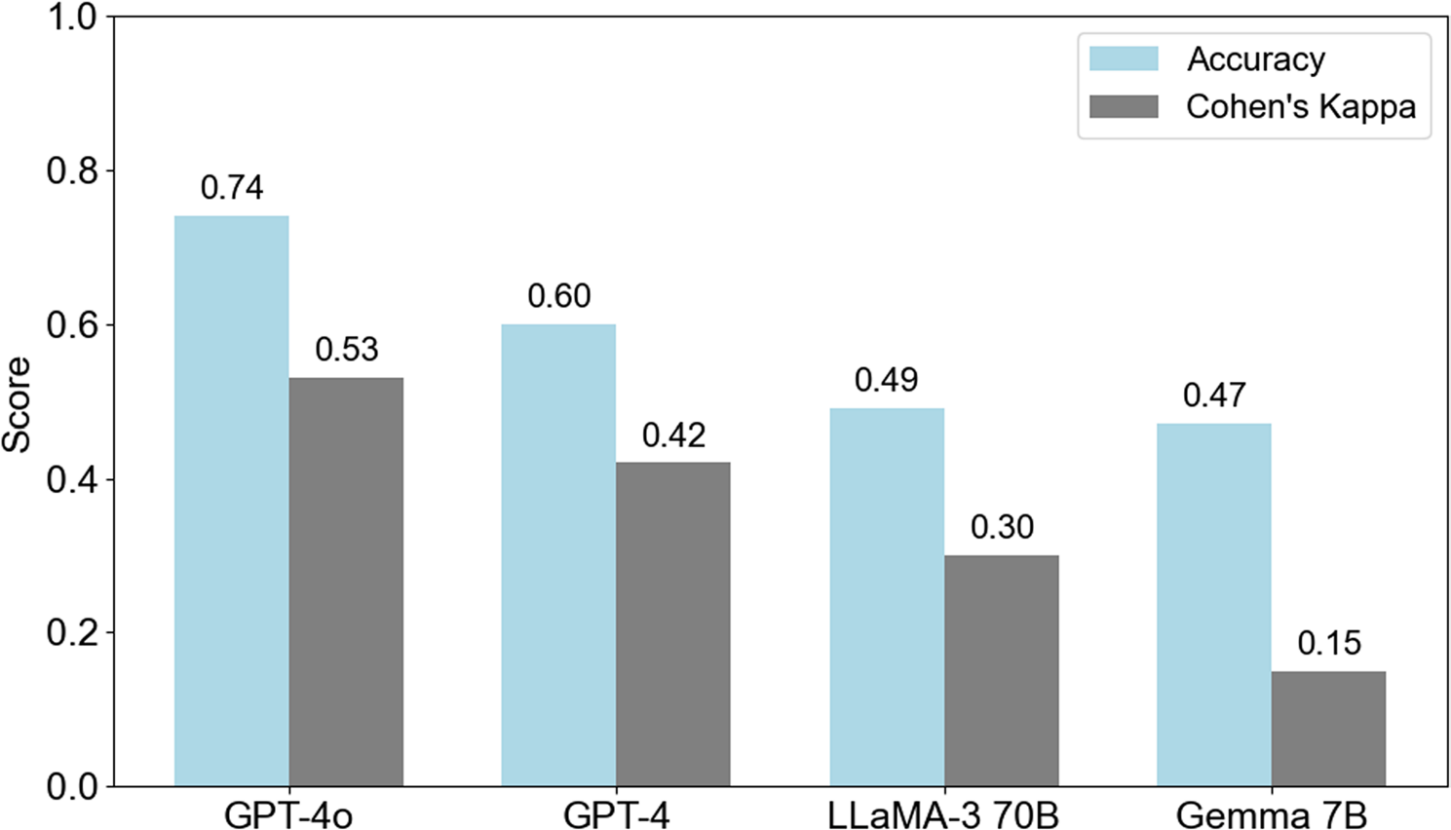
Accuracy and Cohen’s Kappa of LLMs in the classification of the client’s talk type

In comparison with other LLMs, including GPT-4, Llama-3 70B, and Gemma 7B, each model categorized client utterances and showed 0.60, 0.49, and 0.47 accuracy and Cohen’s Kappa of 0.42, 0.30, and 0.15 with expert annotations (Fig. 3.).

The model designs and transition matrices of the estimated final models for the high-quality and low-quality groups were derived from the output sequences of GPT-4 and Llama-3 70B. Although the HMM successfully produced a transition matrix and its corresponding emission distribution based on the output sequence from Gemma 7B, the resulting emission distribution lacked clinical interpretability. Specifically, the inferred emission means did not follow the expected motivational gradient: highest for ‘Toward Change,’ intermediate for ‘Non-Determined,’ and lowest for ‘Away from Change.’ This prevented reliable labeling and interpretation of the latent states. The observed Frobenius norm for differences of the transition matrices and *p*-values of high-quality interview groups between GPT-4o and GPT-4 were 0.21 and 0.92, respectively, while for low-quality interview groups, they were 1.42 and < 0.001. Similarly, the observed Frobenius norm and *p*-values for high-quality interview groups between GPT-4o and Llama-3 70B were 0.75 and < 0.001, respectively, and for low-quality interview groups, 1.46 and < 0.001.

**Fig. 4:**
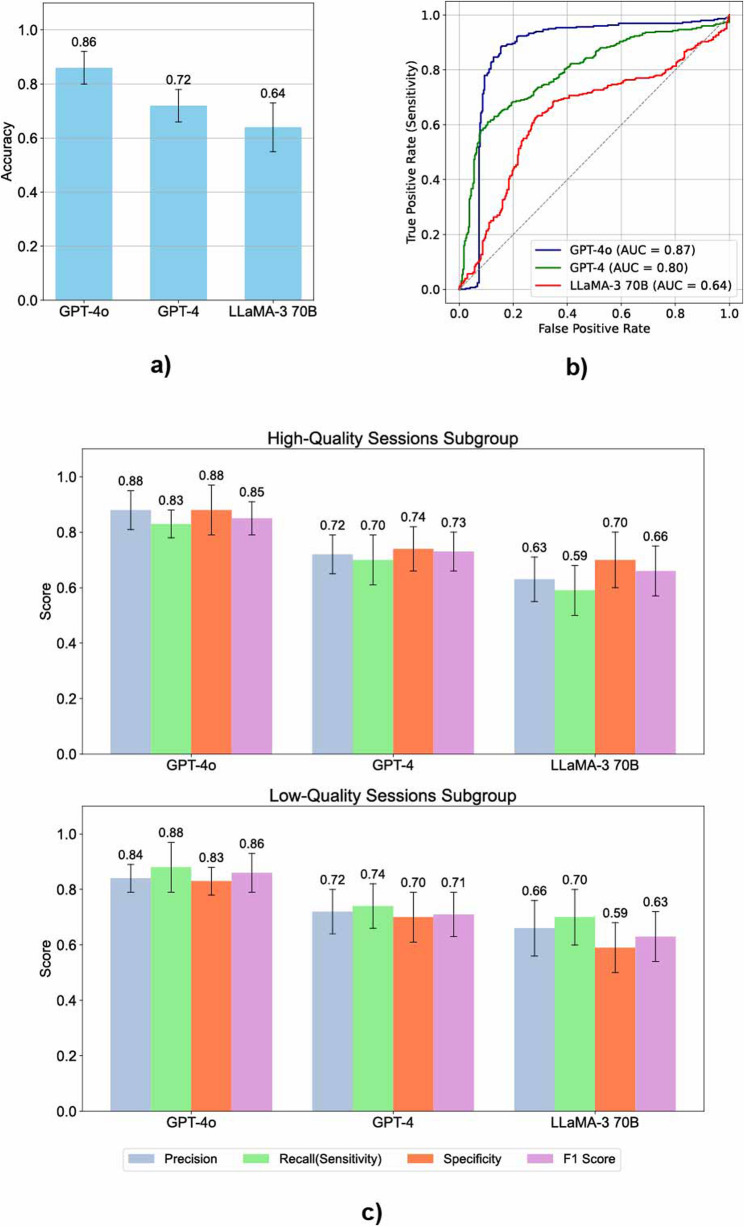
Evaluation of HMM-derived transition feature models for interview quality prediction under a balanced class distribution. (**a**) Mean LOOCV accuracy (± standard deviation) for each model (GPT-4o, GPT-4, LLaMA-3 70B). (**b**) ROC curves with AUC values, summarizing overall classification performance across thresholds. (**c**) Class-wise performance metrics (Precision, Recall, Specificity, and F1 Score) for high- and low-quality session subgroup classification. Error bars represent standard deviations across 10 LOOCV iterations

**Fig. 5:**
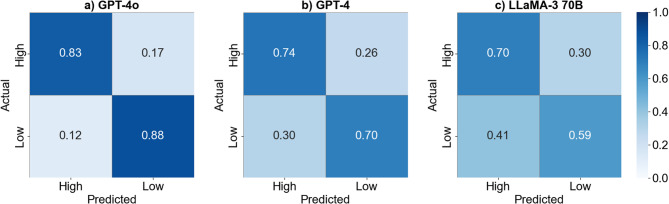
Heatmaps of confusion matrices showing each LLM’s classification performance in predicting MI session quality (high vs. low) under a balanced class distribution. Rows represent true class labels and columns represent predicted labels. Cell values indicate row-wise proportions. Results are shown for (**a**) GPT-4o, (**b**) GPT-4, and (**c**) LLaMA-3 70B

The mean LOOCV accuracies of the logistic regression models trained on HMM-derived features from GPT-4o, GPT-4, and LLaMA-3 70B were 0.86 (± 0.06), 0.72 (± 0.06), and 0.64 (± 0.09), respectively (Fig. 4a). The corresponding mean ROC AUC scores were 0.87 (± 0.05), 0.80 (± 0.06), and 0.64 (± 0.09) (Fig. 4b). For GPT-4o, the performance metrics for high-quality sessions were: precision 0.88 (± 0.07), recall (sensitivity) 0.83 (± 0.05), specificity 0.88 (± 0.09), and F1 score 0.85 (± 0.06). For low-quality sessions, the scores were: precision 0.84 (± 0.05), recall (sensitivity) 0.88 (± 0.09), specificity 0.83 (± 0.05), and F1 score 0.86 (± 0.07). For GPT-4, high-quality session scores were: precision 0.72 (± 0.07), recall 0.70 (± 0.09), specificity 0.74 (± 0.08), and F1 score 0.73 (± 0.07). Low-quality session scores were: precision 0.72 (± 0.08), recall 0.74 (± 0.08), specificity 0.70 (± 0.09), and F1 score 0.71 (± 0.08). For LLaMA-3 70B, high-quality session scores were: precision 0.63 (± 0.08), recall 0.59 (± 0.09), specificity 0.70 (± 0.10), and F1 score 0.66 (± 0.09). Low-quality session scores were: precision 0.66 (± 0.10), recall 0.70 (± 0.10), specificity 0.59 (± 0.09), and F1 score 0.63 (± 0.09). (Fig. 4c.). Confusion matrices illustrating class-specific prediction outcomes for each model are shown in Fig. 5. Subgroups based on the output sequences of Gemma 7B could not be analyzed, as the emission distributions failed to demonstrate clinical relevance, with initial and emission probabilities that were inconsistent with expected clinical patterns.

To test the generalizability of the classification model under conditions that reflect the original data distribution, we repeated the LOOCV analysis using 30 high-quality and 10 low-quality subgroups. GPT-4o achieved a mean accuracy of 0.84 (± 0.09), followed by GPT-4 0.75 (± 0.08) and LLaMA-3 70B 0.72 (± 0.05). The mean ROC AUC scores were 0.84 (± 0.12) for GPT-4o, 0.74 (± 0.09) for GPT-4, and 0.65 (± 0.17) for LLaMA-3 70B. For high-quality sessions, GPT-4o achieved precision of 0.84 (± 0.08), recall of 0.98 (± 0.02), specificity of 0.40 (± 0.34), and F1 score of 0.90 (± 0.05). Corresponding values for GPT-4 were 0.77 (± 0.06), 0.95 (± 0.03), 0.11 (± 0.24), and 0.85 (± 0.05), and for LLaMA-3 70B were 0.75 (± 0.03), 0.95 (± 0.04), 0.04 (± 0.12), and 0.84 (± 0.03). For low-quality sessions, GPT-4o achieved precision of 0.57 (± 0.47), recall of 0.40 (± 0.34), specificity of 0.98 (± 0.02), and F1 score of 0.47 (± 0.39). GPT-4 yielded values of 0.25 (± 0.40), 0.11 (± 0.24), 0.95 (± 0.03), and 0.14 (± 0.27), while LLaMA-3 70B yielded 0.08 (± 0.24), 0.04 (± 0.12), 0.95 (± 0.04), and 0.05 (± 0.16), respectively. These results are summarized in Supplementary Fig. 1 and Supplementary Fig. 2.

As a baseline comparison, we evaluated a direct LLM-only approach in which GPT-4o was prompted to classify session quality based on the Full transcript. This method yielded a mean accuracy of 0.67 (± 0.02). For high-quality sessions, recall of 0.56 (± 0.02), specificity of 1.00 (± 0.00), and F1 score of 0.72 (± 0.02). For low-quality sessions, the corresponding scores were 1.00 (± 0.00) for recall, 0.56 (± 0.02) for specificity, and 0.60 (± 0.01) for F1 score. These results are summarized in Supplementary Fig. 3.

## Discussion

The results of the study reveal that the proposed framework effectively differentiates between high- and low-quality MI sessions as a proof-of-concept. GPT-4o achieved 0.74 accuracy in categorizing client utterances, providing reliable input for HMM analysis. High-quality sessions demonstrated dynamic transitions among motivational states, reflecting active client engagement, while low-quality sessions were characterized by persistent resistance-oriented states, indicating stagnation. A statistically significant difference (*p* < 0.001) between the Frobenius norms of the transition matrices of the high- and low-quality sessions was observed. The significant differences in transition matrices between high- and low-quality sessions underscore the utility of the proposed framework in assessing MI quality. High-quality sessions demonstrated greater flexibility in motivational state transitions, with clients moving fluidly between “Towards Change” and other states. This dynamic engagement is consistent with the goals of MI, which aim to guide clients toward change while addressing ambivalence [6]. In contrast, low-quality sessions exhibited rigid motivational patterns, with a strong persistence in the “Away From Change” state. While individual transition probabilities do not in themselves imply improved client outcomes, the overall transition patterns provide structured, interpretable summaries of motivational dynamics that support automated evaluation of session quality, as demonstrated in our quality prediction analysis.

In comparison with other LLMs, including GPT-4, Llama-3 70B, and Gemma 7B, the quality of results demonstrated a correlation with both model size and optimization capabilities. Models with smaller parameter sizes or less advanced training optimization exhibited lower alignment with expert annotations of client talk types [12]. Among these models, GPT-4 achieved an accuracy of 0.60, while Llama-3 70B, which has fewer parameters than GPT-4 and GPT-4o, showed a reduced accuracy of 0.49. Gemma 7B, the smallest model evaluated, demonstrated the lowest accuracy at 0.47 (Fig. 2).

The transition probability matrices produced by the output sequences of each model also revealed qualitative and statistical differences. Although the transition probability matrix generated by the GPT-4’s output sequence for the high-quality MI group showed a slightly more static motivational trajectory compared to the dynamic transitions in GPT-4o’s matrix, the result did not contradict clinical knowledge. Additionally, a permutation test of Frobenius norms between GPT-4o and GPT-4 output sequences in the high-quality MI group revealed no statistically significant difference (*p* = 0.9150). However, in the low-quality MI group, GPT-4’s transition probability matrix indicated a higher probability of transitioning from “Away From Change” to “Neutral” (0.62) than persisting in “Away From Change” (0.25). This is inconsistent with clinical expectations for low-quality MI sessions. Furthermore, the permutation test showed a significant difference between the transition probability matrices of GPT-4o and GPT-4 in the low-quality MI group (*p* < 0.001). For Llama-3 70B, the transition probability matrix in the high-quality MI group captured some aspects of high-quality sessions, such as strong persistence in “Towards Change.” However, it deviated significantly in its handling of the “Away From Change” state, where the probability of persisting in this state was 0.72. This contradicts the expected dynamics of high-quality sessions. A permutation test confirmed a significant difference between the transition matrices of GPT-4o and Llama-3 70B in the high-quality MI group (*p* < 0.001). Similarly, in the low-quality MI group, Llama-3 70B’s transition matrix aligned with low-quality session characteristics but exhibited limitations in modeling nuanced motivational dynamics. The model overemphasized stagnation in “Neutral” and slightly overestimated transitions to “Towards Change.” The permutation test again indicated a significant difference between the matrices of GPT-4o and Llama-3 70B in the low-quality MI group (*p* < 0.001).

The predictive performance of the logistic regression model trained on HMM-derived features improved with model size. Under the balanced condition (1:1 high- to low-quality subgroup ratio), GPT-4o, GPT-4, and LLaMA-3 70B achieved mean LOOCV accuracies of 0.86, 0.72, and 0.64, respectively (Fig. 4a). ROC analysis further supported this trend, with GPT-4o achieving the highest AUC (0.87), followed by GPT-4 (0.80) and LLaMA-3 70B (0.64) (Fig. 4b). Class-wise analysis revealed that GPT-4o consistently outperformed the other models across both high- and low-quality sessions. (Fig. 4c). These results suggest that larger language models not only improve overall classification accuracy but also yield more stable performance across both clinically meaningful quality categories.

To assess robustness, we also conducted a secondary validation using the original 3:1 class distribution. This analysis yielded mean accuracies of 0.84 for GPT-4o, 0.75 for GPT-4, and 0.72 for LLaMA-3 70B, with corresponding ROC AUC scores of 0.84, 0.74, and 0.65. Despite the imbalance, all models maintained high recall for high-quality sessions (GPT-4o: 0.98; GPT-4 and LLaMA-3 70B: 0.95) and strong F1 scores (GPT-4o: 0.90; GPT-4: 0.85; LLaMA-3 70B: 0.84), indicating that majority-class detection remained stable.

In contrast, performance on low-quality sessions declined sharply, particularly for GPT-4 and LLaMA-3 70B. GPT-4o retained moderate minority-class performance (precision: 0.57; recall: 0.40; specificity: 0.98 F1: 0.47;), while GPT-4 and LLaMA-3 70B exhibited minimal detection capability (precision: 0.25 and 0.08; F1: 0.14 and 0.05). (Supplementary Fig. 1). Confusion matrix analyses (Supplementary Fig. 2) further revealed that while high-quality sessions were reliably classified across all models, recall for low-quality sessions dropped markedly with class imbalance (GPT-4o: 0.40; GPT-4: 0.11; LLaMA-3 70B: 0.04), illustrating the trade-off in minority class sensitivity. These findings suggest that while larger LLMs such as GPT-4o offer superior predictive accuracy and comparatively better resilience to imbalance, overall performance on low-quality sessions remains vulnerable to class distribution. Addressing this limitation may require further advances in LLM design or explicit training-time mitigation strategies to ensure stability across clinically relevant subgroups.”

In addition to evaluating performance across LLM-generated utterance sequences, we conducted a comparative baseline analysis using GPT-4o prompted directly to classify session-level interview quality. This LLM-only approach yielded a mean accuracy of 0.67 (± 0.02), and for high-quality sessions, recall of 0.56 (± 0.02), specificity of 1.00 (± 0.00), and F1 score of 0.72 (± 0.02). For low-quality sessions, it achieved perfect recall (1.00 ± 0.00) with specificity of 0.56 (± 0.02) and F1 score of 0.60 (± 0.01) (Supplementary Fig. 3). In contrast, the LLM + HMM pipeline using GPT-4o achieved substantially better performance: mean accuracy of 0.86 (± 0.06), and balanced classification across both high- and low-quality sessions. These results demonstrate that modeling motivational dynamics via HMMs not only improves predictive accuracy but also leads to more balanced and robust classification across both clinically meaningful quality categories. Also, beyond predictive performance, this structured approach offers greater transparency. Each stage of utterance classification, state inference, and transition modeling can be inspected independently, allowing for interpretable and clinically meaningful assessments of client engagement over time. This modularity also reduces reliance on prompt-tuned reasoning and improves reproducibility.

These findings align with scaling laws observed in LLMs, which suggest that performance on complex tasks, including MI, improves as model size and training data increase [32–34]. Larger models, like GPT-4o and GPT-4, are better equipped to handle the nuanced evaluation of utterances in MI sessions due to their enhanced reasoning and natural language understanding capabilities. This trend is supported by our results, which indicate a clear correlation between model size and performance. The observed accuracy, alignment with expert annotations, and mean and standard deviation of LOOCV accuracy in our study suggest that increasing model size improves the model’s ability to assess and classify client utterances in MI sessions effectively.

Building on the promising results of this study, a key future direction involves expanding the framework’s application to real-world counseling scenarios. While this study utilized MI demonstration videos, applying the framework to live counseling sessions could provide valuable insights into its robustness under more dynamic and variable conditions. Real-world interactions often include overlapping or contradictory motivational signals, as well as external factors such as time constraints or client distractions. Testing the framework in such settings would not only validate its adaptability but also enhance its utility in diverse therapeutic contexts. Another direction for future research involves integrating this framework with other counseling techniques. While this study focused on MI, the principles underlying the framework—the quantification of hidden states and their transitions—are broadly applicable to other therapeutic modalities. For instance, cognitive-behavioral therapy (CBT) could benefit from similar analyses, modeling transitions between cognitive distortions and adaptive thought patterns. Exploring these applications could significantly expand the framework’s utility and contribute to the broader field of psychotherapy research.

One of the most promising implications of this framework lies in its potential for real-time application. While our current pipeline operates retrospectively, its sequential, utterance-level design simulates a real-time streaming scenario. This suggests that, with further development, the model could be extended to support live motivational state monitoring during MI sessions. However, several key components necessary for real-time decision-making remain absent. Notably, the model does not yet account for the timing of interventions, the optimal “dosage” of influence (e.g., how strongly to guide toward change), or the context-sensitive adaptation of therapist strategies based on motivational trajectories. These capabilities would be essential for translating motivational state tracking into actionable feedback or guidance. Future work may explore sequential decision-making frameworks, such as reinforcement learning or policy modeling, to determine when and how to intervene optimally based on evolving client states. We acknowledge that real-time application remains an aspirational goal, and substantial work is needed to validate and operationalize this framework in live clinical settings.

Additionally, the framework could serve as a valuable tool for objectively comparing the quality of MI sessions conducted by various virtual counselors. By analyzing motivational state transitions and session dynamics, the framework can benchmark the effectiveness of different virtual models in facilitating behavior change. Such comparisons would enable evidence-based improvements, ensuring that virtual counselors consistently provide high-quality interventions.

Beyond interview quality assessment and training applications, the sequence of hidden states decoded by the HMM holds a potential clinical application for predicting client prognosis. Research has shown that clients’ final motivational states, as inferred by HMMs, are strong indicators of long-term behavioral outcomes. For instance, Bertholet et al. (2010) demonstrated that a client’s state at the end of a brief motivational intervention was significantly associated with alcohol consumption outcomes 12 months later [21]. Clients who ended in a “Towards Change” state consumed significantly less alcohol than those whose final state was “Away From Change,” regardless of their initial state. This finding suggests that the trajectory of motivational states during an intervention, especially the concluding state, could serve as a valuable predictor of treatment efficacy. In our study, the ability to decode state sequences and identify final states offers a similar opportunity to evaluate prognosis. For instance, if a client transitions from “Non-Determined” to “Towards Change” by the end of a session, the trajectory may indicate successful engagement and readiness for change, suggesting a positive outlook. Conversely, persistence in or reversion to “Away From Change” could signal the need for alternative interventions or additional support. By incorporating such prognostic insights, the framework could provide counselors with actionable recommendations to tailor follow-up strategies and maximize the likelihood of sustained behavioral change.

Despite its potential, this framework has several limitations that warrant consideration. The relatively small sample size (30 high-quality and 10 low-quality sessions) limits the generalizability of the findings, as it does not fully capture the diversity of populations, settings, or counselor-client dynamics. This limitation, while significant, does not invalidate the results since the observed differences were statistically robust (*p* < 0.001). Nonetheless, future studies should validate these findings with larger and more diverse datasets. Moreover, the source data used in this study consists of publicly available demo interviews, which may be cleaner and more structured than real-world clinical encounters and do not include sessions of ambiguous or intermediate quality. Consequently, additional validation using raw, field-collected MI sessions is necessary to assess the robustness of the framework under more ambiguous and variable conditions. Also, although subgroups were treated as independent units for cross-validation, we acknowledge that partial overlap in session composition across subgroups may have introduced minor dependencies between training and test folds. This design choice was a pragmatic trade-off to ensure sufficient data length for stable HMM training. However, we recognize that it may slightly inflate model performance and are planning future validations using longer real-life interviews that allow for HMM parameter estimation at the individual session level. Also, while we anticipate that real-world motivational interviews will be sufficiently long to support session-level HMM modeling, we acknowledge that variability in session length and utterance density may still pose challenges for stable parameter estimation in some cases and represents an important area for further investigation. Additionally, the reliance on the LLM’s utterance classification, which achieved 0.74 accuracy and a Cohen’s Kappa of 0.52—comparable to IAA presented in the previous studies for similar tasks, leaves room for improvement, particularly in handling ambiguous or mixed utterances. Likewise, the strength scores used in our HMM analysis were generated solely by the LLM without human-provided ground-truth supervision. Although the dataset lacked ground-truth numeric strength labels, the LLM-generated scores were guided by structured MI-based prompts and reviewed by a board-certified psychiatrist. This process ensured clinical plausibility and interpretability of the scores used in the HMM analysis. Interpretability also poses a challenge; translating transition matrices into actionable insights for counselors will require the development of user-friendly visualizations and tools. Lastly, ethical considerations, including data privacy, informed consent, and potential algorithmic bias, must be addressed to ensure the framework’s responsible and equitable application in therapeutic settings [11, 35, 36]. Taken together, these results illustrate the feasibility of applying LLM-HMM models for MI quality assessment but should be interpreted within the context of a proof-of-concept study. Future work must assess the robustness of this framework using naturalistic data collected from real-world clinical practice.

Still, the integration of LLMs and HMMs represents a significant advancement in the field of MI assessment. By providing an objective, data-driven approach, the framework aligns with the increasing emphasis on evidence-based practice in counseling. It offers a scalable solution to the challenges of manual coding and subjective evaluation, paving the way for more consistent and reliable assessments of MI quality. Moreover, the framework’s emphasis on motivational state dynamics complements existing approaches, providing a richer understanding of the factors that drive client progress.

In conclusion, this study demonstrates the feasibility and utility of combining LLMs and HMMs to evaluate MI quality. The ability to quantify motivational state transitions and distinguish between high- and low-quality sessions offers a powerful tool for advancing MI practice. While several limitations remain, the proposed framework lays the groundwork for future innovations in counseling assessment. By addressing these limitations and exploring new applications, this framework has the potential to transform both the practice and training of MI, ultimately enhancing outcomes for clients and practitioners alike.

## Conclusion

The proposed LLM-HMM framework effectively quantified MI interview quality and demonstrated predictive accuracy in assessing groups of interviews. Its performance was influenced by the model size and training data of the LLM, aligning with established scaling laws in AI research. While promising, the current framework remains preliminary and requires validation on real-world counseling data. Future studies should test its applicability in routine clinical settings and expand its use beyond MI to explore broader utility.

## Supplementary Information

Supplementary Material 1.

## Data Availability

The datasets analysed during the current study are available in the AnnoMI repository, https://github.com/uccollab/AnnoMI. The cleaned and structured version of the AnnoMI dataset used in our study are available in https://github.com/kyungholim627/annomi-cleaned-lmmhmm.

## Abbreviations

LLM: Large Language Model
MI: Motivational Interview
HMM: Hidden Markov Model
CBT: Cognitive Behavioral Therapy

## Footnotes

1
https://docs.langchain.com/docs.
